# The Prognostic Value of High Platelet Reactivity in Ischemic Stroke Depends on the Etiology: A Pilot Study

**DOI:** 10.3390/jcm9030859

**Published:** 2020-03-20

**Authors:** Adam Wiśniewski, Karolina Filipska, Joanna Sikora, Robert Ślusarz, Grzegorz Kozera

**Affiliations:** 1 Department of Neurology, Faculty of Medicine, Nicolaus Copernicus University in Toruń, Collegium Medicum in Bydgoszcz, 85-094 Bydgoszcz, Poland; 2 Department of Neurological and Neurosurgical Nursing, Faculty of Health Sciences, Nicolaus Copernicus University in Toruń, Collegium Medicum in Bydgoszcz, 85-821 Bydgoszcz, Poland; karolinafilipskakf@gmail.com (K.F.); robert_slu_cmumk@wp.pl (R.Ś.); 3 Experimental Biotechnology Research and Teaching Team, Department of Transplantology and General Surgery, Nicolaus Copernicus University in Toruń, Collegium Medicum in Bydgoszcz, 85-094 Bydgoszcz, Poland; joanna.sikora@cm.umk.pl; 4 Medical Simulation Centre, Medical University of Gdańsk, Faculty of Medicine, 80-210 Gdańsk, Poland; gkozera1@wp.pl

**Keywords:** ischemic stroke, platelet reactivity, aspirin resistance, large vessel disease, carotid stenosis, clinical outcome, prognosis

## Abstract

Background: Reduced aspirin response may result in a worse prognosis and a poor clinical outcome in ischemic stroke. The aim of this prospective pilot study was to assess the relationship between platelet reactivity and early and late prognosis, and the clinical and functional status in ischemic stroke, with the role of stroke etiology. Methods: The study involved 69 subjects with ischemic stroke, divided into large and small vessel etiological subgroups. Platelet function testing was performed with two aggregometric methods—impedance and optical—while the clinical condition was assessed using the National Institute of Health Stroke Scale (NIHSS) and the functional status was assessed using the modified Rankin Scale (mRS) on the first and eighth day (early prognosis) and the 90th day of stroke (late prognosis). Results: The initial platelet reactivity was found to be higher in patients with severe neurological deficits on the 90th day after stroke, than in the group with mild neurological deficits (median, respectively, 40 area under the curve (AUC) units vs. 25 AUC units, *p* = 0.033). In the large vessel disease group, a significant correlation between the platelet reactivity and the functional status on the first day of stroke was found (correlation coefficient (R) = 0.4526; *p* = 0.0451), the platelet reactivity was higher in the subgroup with a severe clinical condition compared to a mild clinical condition on the first day of stroke (*p* = 0.0372), and patients resistant to acetylsalicylic acid (aspirin) had a significantly greater possibility of a severe neurological deficit on the first day of stroke compared to those who were sensitive to aspirin (odds ratio (OR) = 14.00, 95% confidence interval (CI) 1.25–156.12, *p* = 0.0322). Conclusion: High on-treatment platelet reactivity in ischemic stroke was associated with a worse late prognosis regardless of the etiology. We demonstrated a significant relationship between high platelet reactivity and worse early prognosis and poor clinical and functional condition in the large vessel etiologic subgroup. However, due to the pilot nature of this study, its results should be interpreted with caution and further validation on a larger cohort is required.

## 1. Introduction

Stroke is a leading cause of disability and death worldwide and is associated with a worse quality of life [1]. Antiplatelet therapy is used to reduce the risk of recurrent ischemic stroke [2]. Acetylsalicylic acid (aspirin) is a primary antiplatelet agent; however, its effect can vary in different patients [3]. In some patients, a reduced aspirin response may be observed, resulting in a failure to inhibit the platelet reactivity [4]. Platelet function testing can evaluate the effectiveness of aspirin in decreasing platelet aggregation and activation. High on-treatment platelet reactivity or biochemical aspirin resistance is a multifactorial, negative feature that is associated with insufficient antiplatelet therapy [5].

One of the better-understood causes of cerebral ischemia is the pathology of large pre-cranial vessels, most often the internal carotid artery, which accounts for approximately 20–30% of all causes of stroke [6]. Our previous papers demonstrated the hyperaggregation and hyperactivation of platelets in this etiological subtype of ischemic stroke [7,8]. Furthermore, we hypothesize that it may be related to aspirin resistance and affect the clinical condition and prognosis due to the reduced inhibition of platelets. In the next step, we estimate the role of high on-treatment platelet reactivity for the clinical evaluation and prognosis of stroke patients.

Previous reports regarding the relationship between high platelet reactivity and clinical deterioration did not present clear conclusions [9,10,11,12]. The researchers did not focus on the potential role of stroke etiology for significant correlations in this field. The main objective of this study was to determine the relationship between platelet reactivity in the acute phase of ischemic strokes in patients treated with acetylsalicylic acid and the clinical and functional condition of patients, as well as early and late prognosis, with a particular emphasis on cerebral ischemic etiopathogenesis.

## 2. Materials and Methods

### 2.1. Study Population

The perspective, single-center, observational study was conducted at the Department of Neurology at the University Hospital No. 1 in Bydgoszcz. We consecutively enrolled 69 patients between February 2016 and December 2017 who underwent ischemic stroke according to the updated definition of stroke by the American Heart Association/American Stroke Association [13]. All subjects received a standard dose (150 mg) of acetylsalicylic acid based on the current guidelines. We divided the enrolled subjects into two subgroups considering the etiology of ischemic strokes. For the large vessel disease subgroup, we included patients with at least 50% of a carotid artery stenosis on the site correlated with clinical symptoms that were confirmed in an ultrasound examination [14]. The second etiological subgroup, small vessel disease, consisted of subjects with clinical and radiological features related to small vessel disease. We included patients with classic lacunar syndromes (pure motor or sensory stroke or ataxic hemiparesis) and typical neuroimaging markers (small subcortical infarcts <2 cm, hyperintensities in the white matter, lacunes < 15 mm, prominent perivascular spaces, microbleeds, and brain atrophy), where acute ischemic infarcts in neuroimaging were related to clinical symptoms of stroke [15]. The exclusion criteria were: a subject’s inability to make an informed signature (speech disorders, or quantitative or qualitative disturbances of consciousness), an embolic background of ischemic stroke, a previous history of stroke, the chronic use of acetylsalicylic acid before stroke onset, gastrointestinal or urinary bleeding within the last 2 years, low platelet count <100,000/µL, anemia (hemoglobin <9 g/dL), or low hematocrit <35%, “silent” infarcts (infarcts in neuroimaging that are not related to clinical symptoms of stroke). 

### 2.2. Clinical Outcome

Both the clinical status and functional status were assessed within 24 h after admission (first day) to the hospital, on the eighth day of hospitalization (early prognosis), and on the 90th (+/− 5 days) day (late prognosis) after the stroke onset. The clinical status and functional status were assessed using standardized research tools, the National Institute of Health Stroke Scale (NIHSS) and modified Rankin Scale (mRS), respectively. When analyzing the severity of the neurological deficit, two subgroups of patients with stroke were distinguished: a subgroup with a mild neurological deficit (0–5 points on the NIHSS) and a subgroup with moderate and severe neurological deficits (≥6 points on the NIHSS). Regarding the functional status of the patients, two subgroups of stroke patients were distinguished: a favorable prognosis (on the mRS 0–2 points) and an unfavorable prognosis (on the mRS 3–5 points). A comparison of the clinical and functional conditions in both etiological subgroups of the subjects is presented in Table 1.

### 2.3. Ethics Statement

Written informed consent, after revision of the study protocol, was obtained from each participant. This study was approved by the Bioethics Committee of Nicolaus Copernicus University in Torun at Collegium Medicum of Ludwik Rydygier in Bydgoszcz (KB number 73/2016). 

### 2.4. Platelet Function Testing

Aspirin-induced platelet function testing was measured using two methods: optical aggregometry and impedance aggregometry. Blood samples were collected from the participants within 24 h after the stroke onset. To standardize and to unify the time-points of measurements, most cases were performed between 18 and 24 h after the stroke onset, at the same time of day (10–12 AM). The optical aggregometry or light transmission aggregometry (LTA) was performed with an aggregometer (Chrono-Log Corp., Havertown, PA, USA) and the results were expressed as area under the curve (AUC) units. Values over 115 AUC units were defined as high on-treatment platelet reactivity or aspirin resistance. We performed impedance aggregometry using the Multiplate^®^ platelet function analyzer (Roche Diagnostics, France) and its results were expressed as AUC units. For the aspirin-resistant group, we enrolled subjects with values over 40 AUC units. The procedures for performing platelet function testing were similar as described in the previous studies [16,17]. Of our 69 subjects, 43 underwent optical aggregometry measurements, and all 69 subjects underwent impedance aggregometry assessment.

### 2.5. Statistical Analysis

STATISTICA 13.1 (Dell Inc., Round Rock, TX, USA) was used to perform all statistical evaluations. The non-parametric Mann–Whitney U test was used to compare continuous variables. Categorical variables were compared with a Chi-squared test. Spearman’s rank test was used to evaluate the correlations between the variables. The influence of platelet reactivity levels on stroke severity was performed with logistic regression analysis. In the present study, the statistical significance was defined as *p* < 0.05.

## 3. Results

### 3.1. All Subjects

There was no correlation between the platelet reactivity, assessed by Multiplate^®^ and LTA methods, and the severity of the neurological deficit assessed using the NIHSS and functional status of the patients assessed on the mRS in the whole study group (Table 2). The comparison of the severity of neurological deficit (NIHSS) in patients with stroke assessed on the first, eighth, and 90th day after the stroke onset did not show significant differences between the subgroups of patients resistant and sensitive to aspirin (on the first day *p* = 0.8663, on the eighth day *p* = 0.9234, and on the 90th day *p* = 0.8225). There were no differences between the above groups regarding the functional status (mRS) of patients (on the first day *p* = 0.9808, on the eighth day *p* = 0.4610, and on the 90th day *p* = 0.5892).

**Figure 1:**
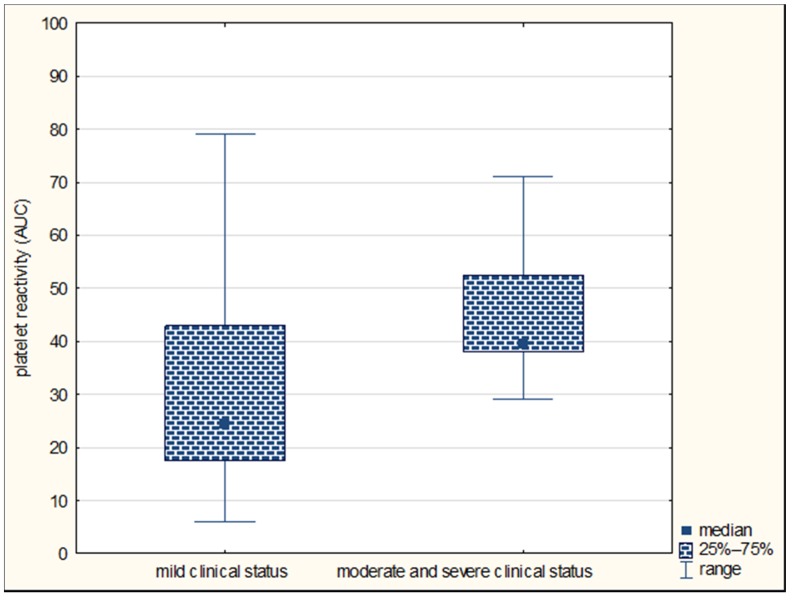
Comparison of platelet reactivity by Multiplate^®^ (in area under the curve (AUC) units) in subgroups of patients with mild and moderate/severe neurological deficits on the 90th day in the general population of stroke patients.

In the present study, we found that the initial platelet reactivity assessed by Multiplate^®^ was higher in patients with moderate/severe neurological deficits compared to a mild deficit on the 90th day after the stroke onset (median, respectively, 40 AUC units vs. 25 AUC units, *p* = 0.033) (Figure 1).

However, there were no differences in the platelet reactivity between the groups distinguished on the basis of the severity of the deficit on the first and eighth day of stroke (on the first day *p* = 0.6599; on the eighth day *p* = 0.3271). The platelet reactivity assessed by Multiplate^®^ did not differ between patients with a favorable and unfavorable prognosis on the first (*p* = 0.6455), eighth (*p* = 0.6744), and 90th day of the disease (*p* = 0.7414). The analysis of the relationship between the platelet reactivity in the LTA method and the clinical and functional status of stroke patients in the whole group of subjects showed no significant relationships (*p* > 0.05).

Logistic regression analysis showed that in the whole group of patients with stroke, aspirin-resistant subjects were 5.5 times more likely to have a severe neurological deficit on the 90th day of stroke than patients who were sensitive to aspirin; however, these differences did not reach statistical significance (odds ratio (OR) = 5.52, 95% confidence interval (CI) 0.54–56.86; *p* = 0.1506).

### 3.2. Two Etiological Subgroups

**Figure 2:**
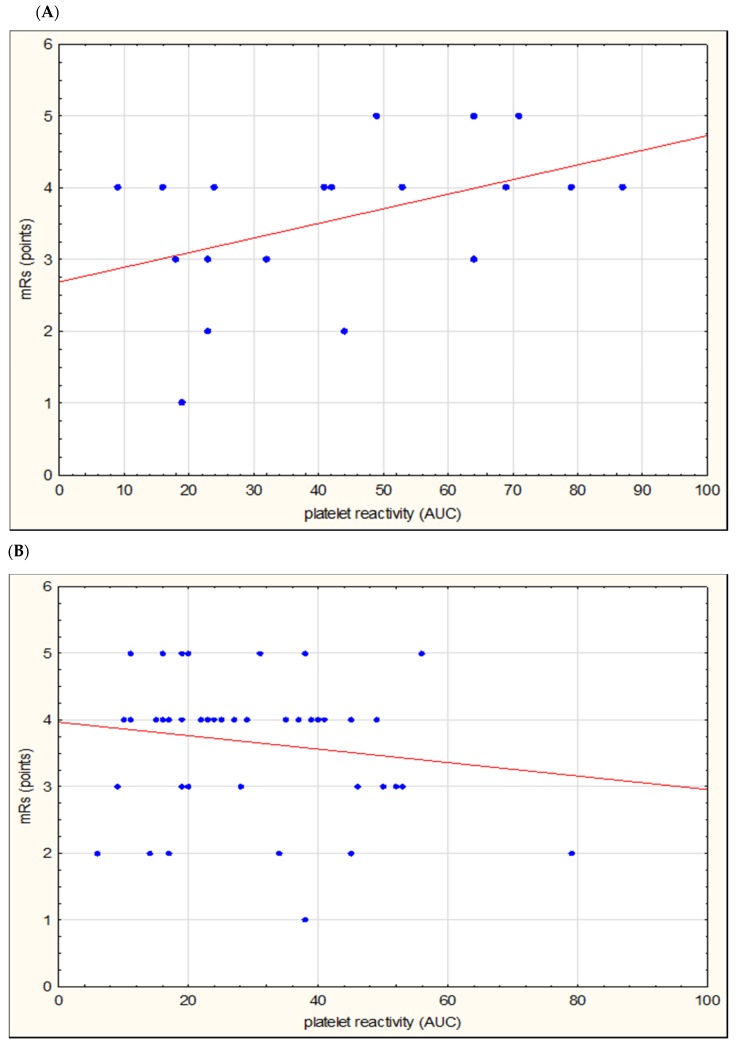
Correlation of platelet reactivity assessed by Multiplate^®^ (in area under the curve (AUC) units) and functional status (modified Rankin scale (mRS) on the first day of stroke) in the subgroup of patients with large vessel disease (**A**) and small vessel disease (**B**).

In the subgroups of patients with the pathology of large and small vessel disease, there were no statistically significant differences in the clinical and functional status of the stroke patients (NIHSS and mRS) (Table 1). In the subgroup of patients with large vessel disease, a significant correlation was found between the platelet reactivity assessed by Multiplate^®^ and the functional status (mRS) on the first day of stroke (correlation coefficient (R) = 0.4526; *p* = 0.0451) (Figure 2, Table 2). 

**Figure 3:**
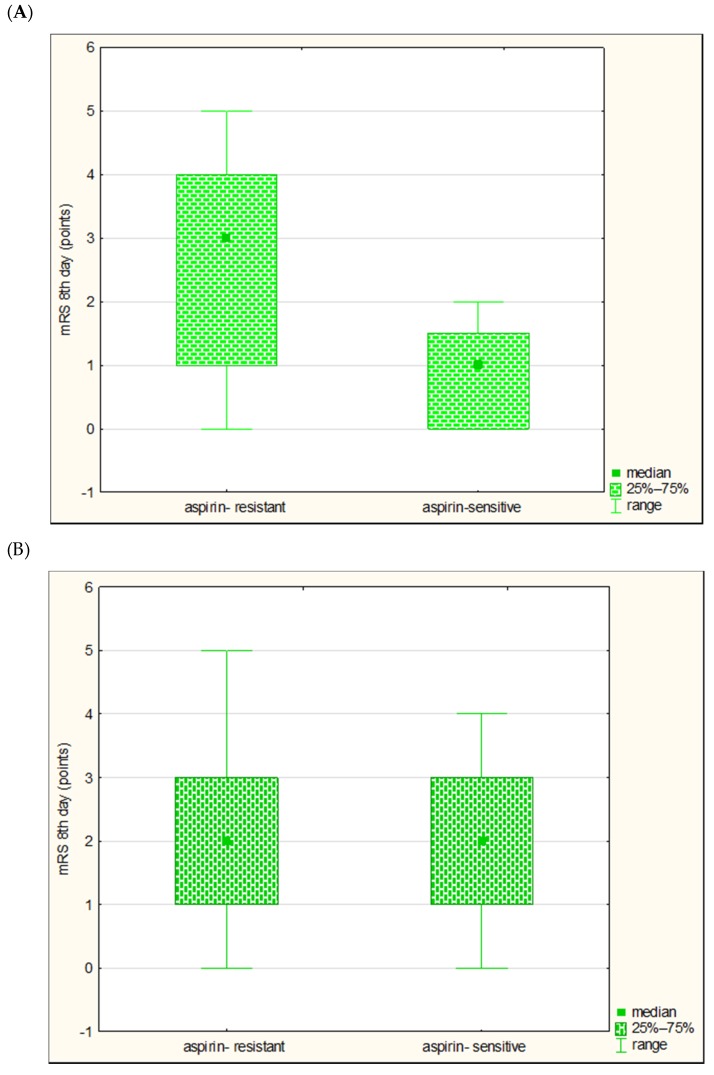
Comparison of the functional conditions (modified Rankin Scale (mRS)) on the eighth day of stroke in aspirin-resistant and aspirin-sensitive subjects in large vessel disease subgroup (**A**) and small vessel disease subgroup (**B**).

Assessing the relationship between the aspirin resistance groups and the severity of clinical deficit in patients with large vessel disease, we found that the aspirin-resistant patients did not differ in the NIHSS scores from aspirin-sensitive patients (on the first day *p* = 0.06, on the eighth day *p* = 0.1167, and on the 90th day *p* = 0.0986). Assessing the relationship with the functional status in patients with large vessel disease, we found that patients with aspirin resistance achieved a higher median of points on mRS on the eighth day of the disease than patients sensitive to aspirin (*p* = 0.0352) (Figure 3). 

There were no differences in the mRS scores on the first and 90th day of stroke (respectively, *p* = 0.0523 for the first day, *p* = 0.0631 for the 90th day). In the subgroup of patients with the pathology of small vessels there were no significant differences in the severity of the clinical deficit and the functional status between the groups distinguished on the basis of the presence or absence of aspirin resistance (*p* > 0.05). 

**Figure 4:**
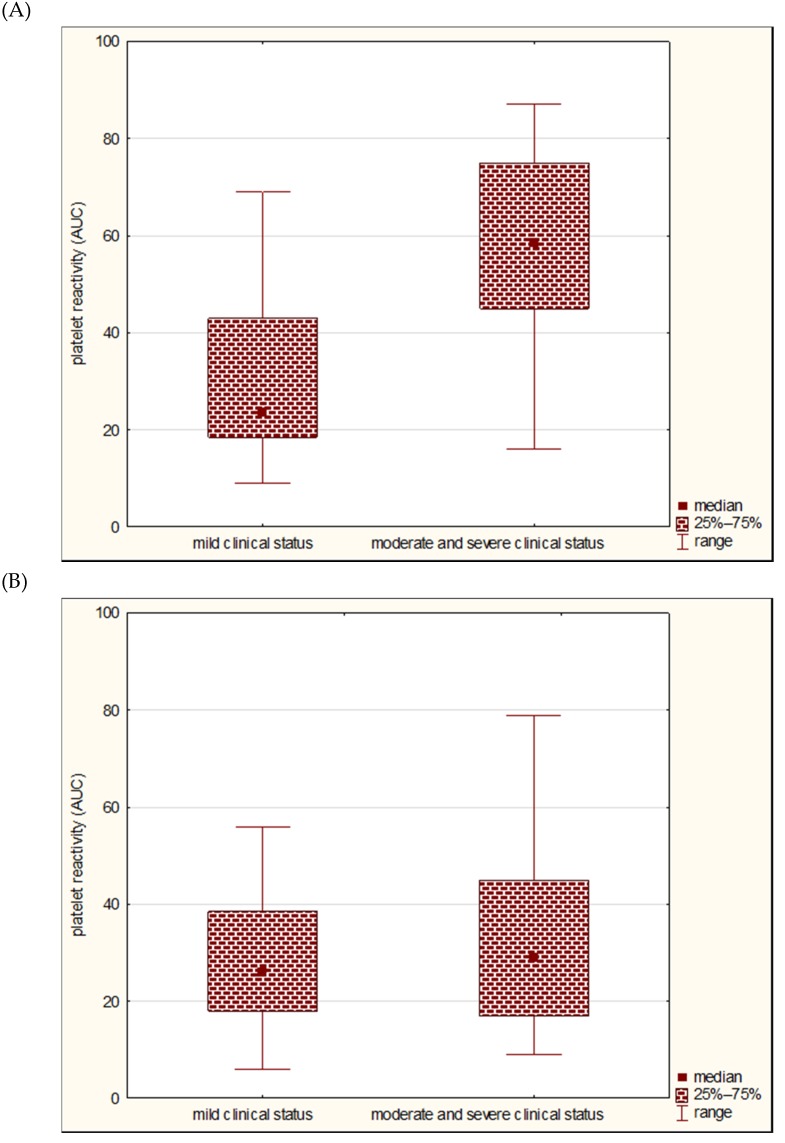
Comparison of platelet reactivity by Multiplate^®^ (in AUC units) in the subgroup of patients with mild and moderate/severe neurological deficits on the first day of stroke in large vessel disease subgroup (**A**) and small vessel disease subgroup (**B**).

Comparing the platelet reactivity in patients with moderate/severe (NIHSS ≥6 points) and mild neurological deficits (NIHHS <6 points), we found that in the subgroup of patients with the pathology of large vessels, the median of platelet reactivity in the Multiplate^®^ method was higher than in the subgroup of patients with severe neurological deficit compared to mild deficit on the first day of the disease (respectively, median 58.5 vs. 23.5 AUC units; *p* = 0.0372) (Figure 4); this did not differ on the eighth day (*p* = 0.0762), on the 90th day (*p* = 0.0982), or on particular days in the subgroup of patients with the pathology of small vessels (*p* > 0.05).

Comparing the platelet reactivity in patients with favorable and unfavorable prognosis, the median of platelet reactivity in Multiplate^®^ method did not differ between the above-mentioned groups both on the first, eighth, and 90th day of stroke in both subgroups (*p* > 0.05).

Logistic regression analysis showed that in the subgroup with large vessel disease, aspirin-resistant subjects had a 14 times greater probability of a severe neurological deficit on the first day of stroke than subjects sensitive to aspirin (OR = 14.00, 95% CI 1.25–156.12, *p* = 0.0322).

## 4. Discussion

In the present study, we demonstrated a significant role of ischemic stroke etiology for the prognostic value of high on-treatment platelet reactivity. We underline that the association between aspirin resistance and poor early clinical and functional conditions in ischemic stroke depends on the etiological subtype of the stroke. In the large vessel disease subgroup, we found a higher platelet reactivity in patients with severe neurological deficits on the first day of stroke, and that aspirin-resistant patients have a significantly higher probability of a severe clinical condition compared to aspirin-sensitive patients. 

The division of patients according to the etiopathogenesis of stroke revealed a significant effect of platelet reactivity on the early functional condition. There was an average, though significant, correlation between platelet reactivity and functional status assessed on mRS on the first day of stroke, and patients resistant to aspirin had higher scores on the mRS (worse prognosis) on the eighth day of stroke than patients who were sensitive to aspirin. Both were present only in the large vessel disease subgroup. No similar results were demonstrated in the whole study population or in the small vessel disease subgroup. 

These novel findings emphasize the great impact of stroke etiology on the association of high on-treatment platelet reactivity and poor early prognosis. However, it is difficult to refer the results of this research with other publications due to the heterogenous populations of the studied groups and different methodologies, and the lack of references in the literature on the impact of stroke etiopathogenesis on the relationship of platelet function testing with the prognosis of stroke subjects. Other authors did not assess the effect of stroke etiopathogenesis on the relationship of platelet reactivity with clinical and functional conditions.

In this study, there was no correlation between the platelet reactivity and the clinical condition in the whole group of subjects using the NIHSS on the first, eighth, and 90th day of stroke. In addition, the division into aspirin-resistant and -sensitive patients did not significantly differentiate the clinical status on particular days of the disease. Only the division of patients with mild to moderate/severe neurological deficits (according to the NIHSS score) revealed a significant effect on a higher initial platelet reactivity (within 24 h after onset) on the more severe clinical conditions, but this relationship was recorded only on the 90th day of stroke. Numerous authors showed that the aspirin-resistant group was characterized by a more severe early clinical condition, assessed on the NIHSS on the first day, than the group sensitive to aspirin [9,10,11,12]. Cheng et al. [12] indicated a significant correlation (R = 0.56) between platelet reactivity and the severity of neurological deficits assessed in the NIHSS. These results led to the conclusion that excessive platelet reactivity and aspirin resistance are associated with a worse clinical condition of patients in the acute phase of stroke. A different observation was demonstrated by Kim et al. [18] and Lai et al. [19], whose studies did not show the effect of platelet reactivity on the severity of the clinical condition assessed on the first day using the NIHSS. Similarly, Englyst et al. [20] did not find significant differences in the clinical status assessed on the NIHSS on the third day of stroke between the groups of patients resistant and sensitive to aspirin. The few literature reports on the long-term clinical conditions assessed on the 30th and 90th days of stroke showed contradictory results. Yip et al. [21] reported that aspirin-resistant patients were characterized by a worse clinical condition (NIHSS) both on the 30th and 90th days than patients sensitive to aspirin, while Lai et al. [19] did not find differences in the clinical condition (NIHSS) between these groups on the 90th day. We hypothesize that the contradictory conclusions from the presented studies may have resulted from omitting the important role of stroke etiology, as demonstrated in the current study.

The impact of platelet reactivity on the functional status of stroke patients is also debatable. Lai et al. [19] suggested a lack of relationship between platelet reactivity and early prognosis, as they did not reveal significant differences on the first day of stroke on mRS in the groups of patients sensitive and resistant to aspirin. On the other hand, Englyst et al. [20] showed a worse functional status of patients evaluated on mRS on the third day of stroke in aspirin-resistant patients compared to the aspirin-sensitive group (mRS median, respectively, 4 vs. 2, *p* = 0.013). Similar conclusions were reached by Sobol et al. [22] (mRS median 3 vs. 2, *p* = 0.02) assessing the functional status of patients on the 10th day of stroke. In our study, in the whole study population, there was no effect of platelet reactivity on the early prognosis—the functional status—on the first and eighth day of stroke or significant differences in the functional status of patients resistant and sensitive to aspirin. The differences in the results of this study from the data presented by other authors may be a result of including all etiological types of stroke (e.g., embolic) and other methodologies (Englyst et al., hemostatic thromboelastography; Sobol et al., Platelet Function Assay (PFA-100)). In addition, according to the results of Amy et al. [23] and Wilterdink et al. [24], aspirin administration before ischemic stroke onset results in a better prognosis of patients evaluated on mRS. Englyst et al. [20] assessed only patients treated with aspirin (for at least three days) before the incident. In the study by Sobol et al. [22], there is no information on whether patients with stroke had previously taken aspirin or only from the first day of stroke. In the only paper regarding late prognosis, Lai et al. [19] showed that on the 90th day of stroke, in the group with aspirin resistance, subjects with a worse functional status were more often reported, rated on mRS at 3–5 points, than in the group sensitive to aspirin (*p* = 0.037). However, the current study did not show any significant effects of platelet reactivity on the late functional status (even considering the etiopathogenesis of stroke). It is worth noting that Lai et al. evaluated the platelet reactivity with the PFA-100 method and recruited patients with stroke who received a dose of 100 mg of aspirin at least five days before platelet reactivity testing, which may have resulted in different functional outcomes than in this study. 

The present study and previous reports, despite various methodologies and inclusion and exclusion criteria of the study population, underline that high on-treatment platelet reactivity may have a negative impact on early and late prognosis and a significant association with poor clinical and functional outcomes in stroke subjects. The novelty demonstrated in this research emphasizes that stroke etiology may be a key factor for the above dependencies. 

According to the results obtained in this study, carotid artery stenosis appears to be an essential platelet activating factor. Tsai et al. [25], who assessed platelet function using flow cytometry, also demonstrated significantly higher platelet aggregation in patients with large vessel pathology compared to small vessel pathology. Importantly, the study was conducted on a similar population of patients with cerebral ischemia to this study (i.e., they excluded patients with stroke due to the embolic background. Similar results were presented by Zheng et al. [11], Kinsella et al. [26], and Dawson et al. [27], who demonstrated that platelet reactivity assessed by different methods is significantly elevated in patients with carotid artery stenosis. Kinsella et al. [28], using the PFA-100, reported that surgical treatment (e.g., stenting) in stroke subjects due to a carotid artery stenosis was associated with a significant reduction of platelet activation. These results were consistent with our current findings, highlighting the role of large vessel disease etiology for ischemic stroke in increasing platelet reactivity. These results indicate that platelet function monitoring may be useful for stroke subjects due to carotid artery stenosis. Additionally, platelet-function-guided individualized antiplatelet therapy can be essential to optimize clinical outcomes and to improve the functional status. 

Unfortunately, both the American and European guidelines for the treatment and prevention of stroke do not distinguish between antiplatelet therapy and stroke pathomechanisms. Regardless of whether it is lacunar stroke or stroke due to a pathology of large extracranial vessels, aspirin administration is recommended for all patients with thrombotic stroke [2]. The current guidelines do not address the issue of aspirin resistance. It seems that this may be due to the lack of large, randomized clinical trials that could be used to develop clear guidelines. 

As stroke in large extracranial pathology accounts for a fairly significant proportion of all strokes, and the results of current and previous studies highlight the significant impact of carotid artery pathology on platelet reactivity relationships with worse clinical conditions and prognoses, we recommend routinely determining platelet reactivity and detecting aspirin resistance, especially in cases of recurrent ischemic events. The authors believe that this would allow for personalized antiplatelet treatment based on platelet function testing, whose effectiveness for this group of patients is a priority.

The authors are aware that this study has several limitations. The evaluation of platelet function was performed only once and at different times during the first 24 h after the onset of stroke and at different times after the first dose of acetylsalicylic acid. It could have contributed to the variations in the measurements of platelet function. A single measurement with poorly validated methods in light of the marked variability of platelet reactivity that was previously demonstrated, may not be sufficient to properly assess the effect of high on-treatment platelet reactivity on the clinical condition and prognosis in ischemic stroke. More work is essential to sequentially determine platelet reactivity on successive days. Another limitation is that biochemical resistance does not always correspond with clinical resistance. The sample size in the study was small and imbalanced between the two etiological subgroups. The lack of recruitment of patients with severe stroke (especially those with impaired consciousness), due to the inability to obtain informed consent, constitutes a huge limitation of the study. Despite using stringent inclusion and exclusion criteria, in the face of low rates of in- hospital atrial fibrillation detection, there is a possibility that a small percentage of subjects may have had another etiology of stroke, such as embolism. 

## 5. Conclusions

This pilot study demonstrated that high on-treatment platelet reactivity is associated with a worse late prognosis in ischemic stroke. In patients with large vessel disease, high platelet reactivity is associated with a worse early prognosis and clinical and functional condition of patients in the acute phase of stroke. The role of etiology demonstrated in this paper is novelty. However, due to the pilot nature of this study, the obtained results should be interpreted with caution. Further research, performed on larger sample size, is essential to validate and confirm our findings and to determine the optimal and personalized antiplatelet therapy. 

## Figures and Tables

**Table 1:** A comparison of the anthropometric data, platelet reactivity, clinical, and functional status in patients with stroke in both etiological subgroups.

Parameter	Large Vessel Disease *n* = 20	Small Vessel Disease *n* = 49	*p*-Value
Age median (range) *	67 (45–85)	68 (40–89)	0.7761
Male N, (%) **	14 (70%)	21 (42.9%)	0.0408
Platelet reactivity: optical aggregometry (AUC) median (range) *	17.1 (0–208.6)	20.4 (0–154.2)	0.7147
Platelet reactivity: impedance aggregometry (AUC) median (range) *	42 (9–101)	27.5 (6–108)	0.0622
NIHSS 1 day median (range) *	5 (2–17)	5 (1–17)	0.6770
NIHSS 8 day median (range) *	2 (0–10)	2 (0–10)	0.8324
NIHSS 90 day median (range) *	1 (0–8)	2 (0–10)	0.6625
mRS 1 day median (range) *	4 (1–5)	4 (1–5)	0.7304
mRS 8 day median (range) *	1 (0–5)	2 (0–4)	0.4999
mRS 90 day median (range) *	1 (0–4)	2 (0–4)	0.5740

* Mann–Whitney U test, ** Chi-squared calculation. AUC, area under the curve; NIHSS, National Institute of Health Stroke Scale; mRS, modified Rankin scale.

**Table 2:** Correlations of the clinical and functional conditions and platelet reactivity in both methods on individual days of stroke in the general population and in the subgroup of patients with large vessel disease.

	General Population		Large Vessel Disease					
	Multiplate^®^		LTA		Multiplate^®^		LTA	
	R	*p*	R	*p*	R	*p*	R	*p*
NIHSS 1 day	0.0713	0.5603	0.0010	0.9948	0.4908	0.0728	0.0010	0.9947
NIHSS 8 days	0.0473	0.6996	0.1472	0.3462	0.2636	0.2614	0.1472	0.3462
NIHSS 90 days	0.0781	0.5233	0.0859	0.5838	0.2801	0.2017	0.0859	0.5837
mRS 1 day	0.0273	0.8240	0.0170	0.9139	0.4526	0.0451	0.01698	0.9139
mRS 8 days	0.1233	0.3128	0.0781	0.6186	0.4068	0.0750	0.0781	0.6186
mRS 90 days	0.0968	0.4288	0.1099	0.4829	0.3676	0.1108	0.1099	0.4826

Spearman’s rank correlation. R, correlation coefficient; LTA, light transmission aggregometry; NIHSS, National Institute of Health Stroke Scale; mRS, modified Rankin scale.
